# Flexible Composite Electrolyte Membranes with Fast Ion Transport Channels for Solid-State Lithium Batteries

**DOI:** 10.3390/polym16050565

**Published:** 2024-02-20

**Authors:** Xiaojun Ma, Dongxu Mao, Wenkai Xin, Shangyun Yang, Hao Zhang, Yanzhu Zhang, Xundao Liu, Dehua Dong, Zhengmao Ye, Jiajie Li

**Affiliations:** 1 School of Materials Science and Engineering, University of Jinan, Jinan 250022, China; 15552600076@163.com (X.M.); 17663061976@163.com (D.M.); xinwenkai2021@163.com (W.X.); 17860612220@163.com (S.Y.); 18669576201@163.com (H.Z.); 18231895913@163.com (Y.Z.); mse_liuxundao@ujn.edu.cn (X.L.); 2 Department of Chemical and Biological Engineering, Monash University, Clayton, VIC 3800, Australia; dehua.dong@monash.edu

**Keywords:** PVDF-HFP/LLZTO, net-like structure, flexible composite electrolyte, PVEC

## Abstract

Numerous endeavors have been dedicated to the development of composite polymer electrolyte (CPE) membranes for all-solid-state batteries (SSBs). However, insufficient ionic conductivity and mechanical properties still pose great challenges in practical applications. In this study, a flexible composite electrolyte membrane (FCPE) with fast ion transport channels was prepared using a phase conversion process combined with in situ polymerization. The polyvinylidene fluoride-hexafluoro propylene (PVDF-HFP) polymer matrix incorporated with lithium lanthanum zirconate (LLZTO) formed a 3D net-like structure, and the in situ polymerized polyvinyl ethylene carbonate (PVEC) enhanced the interface connection. This 3D network, with multiple rapid pathways for Li^+^ that effectively control Li^+^ flux, led to uniform lithium deposition. Moreover, the symmetrical lithium cells that used FCPE exhibited high stability after 1200 h of cycling at 0.1 mA cm^−2^. Specifically, all-solid-state lithium batteries coupled with LiFePO_4_ cathodes can stably cycle for over 100 cycles at room temperature with high Coulombic efficiencies. Furthermore, after 100 cycles, the infrared spectrum shows that the structure of FCPE remains stable. This work demonstrates a novel insight for designing a flexible composite electrolyte for highly safe SSBs.

## 1. Introduction

The escalating demand for highly secure energy storage systems in wearable electronics and power batteries has underscored the imperative for advancements in lithium batteries [1,2,3,4,5]. SSBs with higher safety can be a promising candidate for a spectrum of issues associated with traditional liquid electrolyte systems [6,7,8,9].

Solid electrolytes are broadly categorized into two primary classes: organic and inorganic [10,11,12]. Generally, inorganic electrolytes, exemplified by oxides such as Li_7_La_3_Zr_2_O_12_ (LLZO), exhibit notable mechanical strength and commendable thermal stability, while the ionic conductivity exceeds 10^−3^ S cm^−1^ at room temperature [13,14,15]. Despite these merits, the inherent rigidity of inorganic electrolytes poses challenges at the electrode–electrolyte interface, resulting in elevated interface resistance [16]. Conversely, polymer electrolytes, typified by materials like polyethylene oxide and polyvinylidene fluoride (PVDF), establish favorable interfacial contacts with diverse electrodes [17]. However, their lithium-ion conductivity at room temperature, approximately ~10^−6^ S cm^−1^, is comparatively low, hindering the advancement of organic polymer electrolytes to a certain extent [18,19,20,21].

A novel approach involves incorporating an inorganic electrolyte as an active filler into an organic polymer electrolyte to formulate a polymer composite solid electrolyte [22,23]. This strategy leverages the flexibility inherent in solid electrolytes, resulting in an improvement in ionic conductivity at room temperature [24]. Recent investigations have demonstrated progress in the development of composite polymer electrolytes (CPE), particularly those incorporating LLZO as an active filler [25]. Such formulations exhibit superior mechanical energy characteristics and enhanced ionic conductivity at room temperature compared to other composite polymer electrolytes of similar compositions [26,27,28].

The method for preparing porous electrolytes typically involves the use of extrusion and casting, resulting in porous electrolyte films through solvent evaporation [29]. Y.L et al. [30] prepared a composite solid electrolyte using a solution casting method. However, the pores obtained using this method are often not sufficiently interconnected and have relatively small diameters, making it difficult to store electrolyte materials within these pores. As a result, the electrolyte is prone to consumption, leading to insufficient stability in cycling performance. In recent years, phase transition methods have gained research significance as a pore-forming technique [31]. This method involves a polymer solution system where the solvent is the continuous phase, undergoing a process that transforms into a swollen solid state. In simple terms, the polymer dissolves in a solvent, solvent A, to form a homogeneous solution. When this solution is mixed with another solvent, solvent B, which is miscible with A but does not dissolve the polymer, phase separation and solidification occur within the homogeneous solution. If the phase separation occurs rapidly, solvent A within the homogeneous solution is quickly replaced by solvent B, resulting in the formation of pores within the solidified polymer [31,32,33].

J.Z et al. [34] utilized the phase transition method to prepare a composite solid electrolyte with vertical microchannels. This electrolyte had a significant thickness, leading to longer lithium ion transport distances and increased impedance. By reducing the thickness of the electrolyte film, irregular three-dimensional interconnected large pores were obtained using the phase transition method, creating abundant polymer-active filler interfaces. Additionally, these interconnected large pores provided storage for gel electrolytes, further accelerating lithium ion conduction in a three-dimensional space [35].

In these CPEs, lithium-ion (Li^+^) transport primarily occurs through a unique pathway within the amorphous region of polymer, encompassing the polymer–filler interface and the active filler [36]. It is always lead to an insignificant increase in ionic conductivity by simply mixing the polymer matrix with ceramic particles due to particle agglomeration and lack of a well-defined ceramic–polymer interface [35,37,38,39]. The isolated ceramic particles within polymer fail to create a lithium-ion conductive network, resulting in low conductivity. Recent studies show that constructing a three-dimensional (3D) net-like structure with LLZO filler can reduce particle agglomeration [40]. However, a substantial improvement in ionic conductivity close to ~10^−4^ S cm^−2^ at room temperature still remains challenging [41]. To address these issues, establishing a 3D packing network through in situ polymerization within the polymer electrolyte is crucial, which ensures continuous lithium-ion conductivity throughout the structure [42,43]. Thus, precisely optimizing the morphology and content of active ceramic fillers plays a crucial role in enhancing the electrochemical and mechanical properties of CPE [44].

Herein, a flexible composite electrolyte composed of pores 3D net-like structure and fast ion channels was developed. The polyvinylidene fluoride-hexafluoro propylene (PVDF-HFP) and lithium lanthanum zirconate (LLZTO, Li_6.4_ A_l0.2_La_3_Zr_1.4_Ta_0.6_O_12_) based pores 3D net-like structure created a well-defined ceramic–polymer interface which give the electrolyte high mechanical strength and improved ion conductivity. The filled PVEC-based electrolyte in the pores can collect ions on the porous walls, further providing a fast ion migration channel which further enhances the ionic conductivity at room temperature. This approach, permeating ionic conductive species within a three-dimensional network, demonstrates superior electrode–electrolyte interface contact compared to conventional composite solid-state electrolytes. The as prepared FCPE exhibits a high ionic conductivity of about 1.21 × 10^−4^ S cm^−1^ and cycle stability for 1200 h for a lithium-symmetrical battery at a current density of 0.1 mA cm^−2^. Importantly, when applied in an all-solid-state Li|FCPE|LiFePO_4_ coin cell, it maintains a high specific capacity of 148.5 mA h g^−1^, even after 100 cycles, demonstrating robust cyclic stability. Furthermore, characterization of the solid electrolyte membrane after 100 cycles and 1200 h using FT-IR spectroscopy revealed that the structure of membrane remains stable. This underscores significant prospects for practical applications in the field of solid-state electrolytes.

## 2. Materials and Methods

### 2.1. Materials

Dimethyl sulfoxide (DMSO, >99.9%), Lithium bis(trifluoromethanesulphonyl)imide (LiTFSI, 99.0%), 4-Vinyl-1,3-dioxolan-2-one (VEC, 99.0%), and 2,2’-Azobis(2-methylpropionitrile) (AIBN, 99.0%) were all purchased from Macklin, Shanghai, China. Li_6.4_ A_l0.2_La_3_Zr_1.4_Ta_0.6_O_12_ solid electrolyte powder (LLZTO), Lithium iron phosphate (LFP, P198-S20), and Carbon nanotube dispersants (CNTs)were purchased from Shenzhen Kejing, Shenzhen, China. Poly (vinylidene fluoride-co-hexafluoropropylene) (PVDF-HFP, KynarFlex2801, Mw = 900,000)

### 2.2. Materials Synthesis

The phase conversion method was used to the preparation of an FCPE with a porous structure. An appropriate quantity of PVDF-HFP was dissolved in dimethyl sulfoxide (DMSO) and magnetically stirred for 60 min at 60 °C to yield a 10 wt% polymer clear solution. Subsequently, LLZTO powder was added to the solution in varying ratios (*x* LLZTO/*y* PVDF-HFP = 0%, 5%, 10%, 15%, denoted as PH*x*L where *x* = 0, 5, 10, 15). The resulting mixture was further stirred for 20 min under vacuum conditions to achieve a homogeneously dispersed slurry. The slurry was then cast onto a smooth glass plate at 40 °C, and the resulting membrane was transferred to a vessel filled with ultrapure water. After 5 min, it was removed and dried in a forced-air oven at 60 °C for 12 h, yielding the PVDF-HFP-LLZTO membrane. All membranes were subsequently transferred to a glovebox. The membranes exhibit a thickness of PH10L approximately 19 μm and a diameter of 19 mm.

For the PVEC-based electrolyte precursor, 2.5 M lithium hexafluorophosphate (LiPF_6_) was added to vinylene carbonate (VEC), along with 0.02 wt% azobisisobutyronitrile (AIBN) as an initiator for polymerization. The solid electrolyte, filled with the PVEC-based electrolyte precursor slurry during the battery loading process into the PH*x*L is denoted as PH*x*LE.

In the preparation of the cathode electrode, lithium iron phosphate (LFP), PVDF-HFP and Carbon nanotube dispersants (CNTs) (the mass ratio of the three is 8:1:1) were mixed and stirred in DMSO for 6 h. The final slurry is poured onto an aluminum foil current collector and dried at 100 °C for 24 h. The active material loading on the aluminum foil current collector is about 2.0 mg cm^−2^.

### 2.3. Characterization

The morphological features of the specimens were assessed through scanning electron microscopy (SEM) utilizing the ZEISS Sigma 300 instrument. Transmission electron microscopy (TEM) and elemental mapping images were acquired using a transmission electron microscope (JEM-2100P, JEOL, Tokyo, Japan). X-ray diffraction (XRD) patterns were captured on the Smart Lab 9KW instrument with Cu Kα radiation. Thermogravimetric analyses (TGA) were executed employing a PerkinElmer STA 6000 analyzer. Fourier-transform infrared (FT-IR) spectroscopy was tested on a Nicolet 6700 spectrometer. Raman experiments were performed at Horiba Labram Evolution and were measured from 1064 nm power excitation.

### 2.4. Electrochemical Characterization

Two meticulously polished stainless steel discs (SS) served as blocking electrodes to encapsulate the composite solid electrolyte, establishing a blocking-type cell. The ionic conductivity of FCPE at room temperature (25 °C) was quantified utilizing electrochemical impedance spectroscopy (EIS) in the spectral range of 0.01 to 10^5^ Hz at room temperature with an alternating amplitude of 10 mV. The ionic conductivity (σ) has calculated using the equation σ = L/(R_b_·S).

The electrochemical window of FCPE was determined by employing the linear sweep voltammetry (LSV) technique, which is obtained using a Li/FCPE/SS cell configuration with a scan rate of 10 mV s^−1^ and a scan range of 0 to 4.8 V. Li/FCPE/LiFePO_4_ coin cells were assembled into all-solid-state batteries, and the LAND CT2001A meter was used to perform a charge–discharge test within the voltage range of 2.5 to 3.65 V at 25 °C.

## 3. Results and Discussion

**Figure 1:**
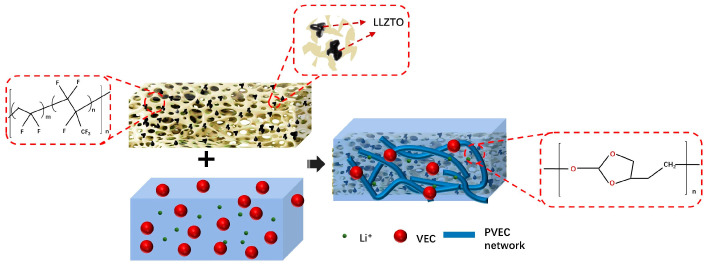
Schematic diagram of the FCPE structure.

**Figure 2:**
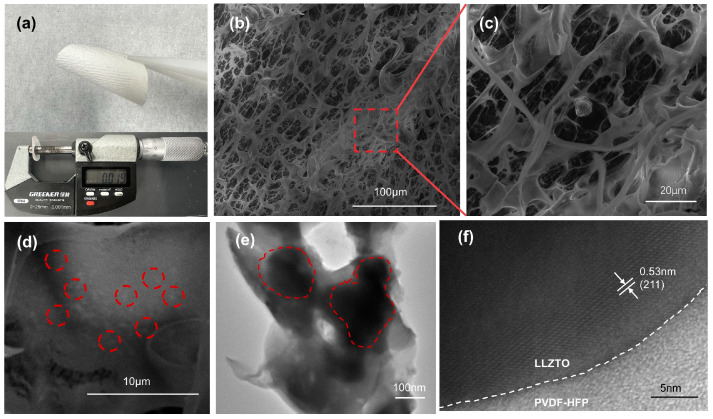
(**a**) Digital image of PH10L; (**b**–**d**) the surface morphology SEM images of PH10L; (**e**) STEM image; and (**f**) high-resolution TEM (HRTEM) image of PH10L.

As depicted in Figure 1, after the phase conversion process, the solid electrolyte exhibits a porous structure with PVDF-HFP encapsulating ceramic powder particles, forming a rich PVDF-HFP-LLZTO interface, constituting a three-dimensional interconnected channel. This porous structure is impregnated with a PVEC-based electrolyte single-ion conductor through in situ polymerization. At the same time, the VEC has not undergone complete polymerization, and free monomers still exist around the polymer chains. The presence of the VEC monomers leads to a reduction in the crystallinity of the polymer, resulting in a shorter polymer chain length, which further promotes the conduction of lithium ions. Figure 2a shows the as-prepared solid electrolyte has a white appearance and flexible characteristics. SEM images revealed that the porous flexible composite electrolyte possesses an interconnected network structure characterized by nest-shaped polymer ducts that are 3D interconnected (Figure 2b–d). The enlarged view of the red rectangular region selected in Figure 2b is shown in Figure 2c, and the enlarged view of Figure 2c is shown in Figure 2d. This distinctive architecture is attributed to the exchange of solvents and non-solvents during phase conversion, driven by a reduction in the Gibbs free energy of the system through the generation of a new phase (β phase) per unit volume from the parent phase (α phase) [45,46]. Among them, white spots on the surface of the film can be further observed in the red dotted circle in Figure 2d. It is believed that the white spots are ceramic particles wrapped by organic matter. The microstructure of the PH*x*L 3D skeleton was observed by TEM. As shown in Figure 2e, the skeleton is a porous structure with a pore diameter of approximately 100 nm. It should be noted that nanoparticles with a diameter of about 200 nanometers were embedded within the skeleton which is consistent with the SEM result in Figure 2e. The interface between the LLZTO phase and the elastomer matrix exhibited a smooth connection, elucidating the achieved structural integrity in the composite electrolyte. Further, high-resolution transmission electron microscopy (HRTEM) images (Figure 2f) showed that the surface of pure LLZTO particles was uniformly coated with amorphous PVDF-HFP. The spacing of the lattice fringes of the particles is 0.53 nm, which is in good agreement with the (211) plane of LLZTO, indicating that the PVDF-HFP coating of LLZTO does not destroy the crystal structure of LLZTO during the phase conversion process [47]. It has been reported that a chemical reaction occurs between PVDF-HFP and LLZTO, ultimately leading to the defluorination of PVDF-HFP [48]. Notably, PVDF-HFP exhibits a propensity for defluorination in alkaline environments, a phenomenon exacerbated by the alkaline nature often exhibited by LLZTO ceramics in various solutions. This study employed a phase transition method. During the transition process, PVDF-HFP is insoluble in water and undergoes rapid solvent exchange upon contact. Uniformly dispersed LLZTO particles are encapsulated within PVDF-HFP, forming numerous large interconnected three-dimensional pores.

**Figure 3:**
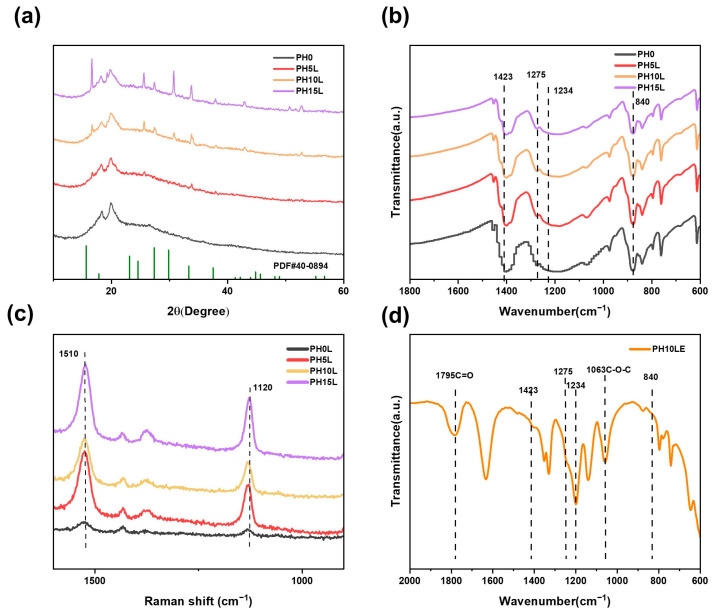
(**a**) XRD curves, (**b**) FT-IR spectrum, and (**c**) Raman spectra of PH0LE to PH15LE at room temperature. (**d**) FT-IR spectrum of PH10LE at room temperature.

The XRD pattern of the composite electrolyte is depicted in Figure 3a. XRD peaks of pure PVDF-HFP film at 20° and 40° reveal the presence of polar β-phase and γ-phase [49]. Upon the incorporation of ceramic fillers, the XRD peaks associated with PVDF-HFP weaken and broaden, indicating a reduction in the crystallinity of the PVDF-HFP component in the electrolyte. Additionally, the introduction of LLZTO corresponds to a cubic garnet crystal structure with the Ia3′d space group, matching the standard peaks of Li_7_La_3_Nb_2_O_12_ (PDF#: 40-0894) [50] well. (The PDF profile of the Li_7_La_3_Nb_2_O_12_ which has a similar cubic phase to LLZTO) [35]. The cubic phase of LLZTO is more favorable for lithium-ion conduction compared to the amorphous phase of PVDF-HFP, contributing to the stability of the composite material. Furthermore, with an increase in the LLZTO content, the characteristic peaks associated with LLZTO gradually intensify. The FTIR spectrum of the electrolyte membrane is depicted in Figure 3b. Vibrational peaks at 840, 1234, 1275, and 1423 cm^−1^ corroborate the XRD results by corroborating the β- and γ-phases of PVDF-HFP [51]. In the Raman spectrum (Figure 3c), as the LLZTO content increases, the peaks at 1120 and 1510 cm^−1^ gradually intensify, likely attributed to C-C stretching vibrations. This indicates that the PVDF-HFP backbone has been modified. This suggests that with the incorporation of LLZTO, the main chain structure of PVDF-HFP undergoes dehydrofluorination reactions [52]. Notably, the FT-IR spectrum of PVEC exhibited prominent peaks corresponding to the C=O bond at 1795 cm^−1^ and the C-O bond at 1063 cm^−1^, indicative of the stability of carbonate units during the thermal polymerization process (Figure S1) [49]. Furthermore, the incorporation of VEC into the framework of PH10L for polymerization results in PH10LE, as shown in Figure 3d. The infrared spectrum of PH10LE exhibits almost identical characteristic peaks to those of PH10L and PVEC, indicating that its structure remains unchanged after the formation of the composite.

**Figure 4:**
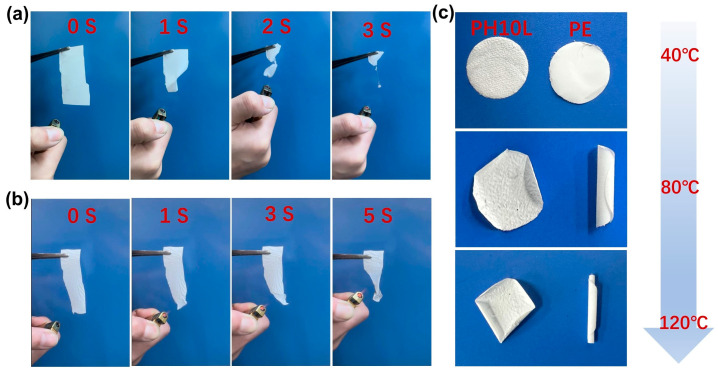
(**a**) Combustion test of commercial PE separator and (**b**) PH10L. (**c**) The digital photos of PH10L and PE separator heated at different temperatures.

The thermal characteristics of the PH0L and PH10L films were elucidated through TGA. As shown in Figure S2, below 400 °C, both pure PVDF-HFP membrane and PH10L remain stable without decomposition. Both experience gradual weight loss and abrupt decomposition around 450 °C, indicating a decrease in their thermal stability. However, the network-like electrolyte film with LLZTO fillers exhibits a 20 °C higher temperature for rapid thermal decomposition compared to pure PVDF-HFP after the addition of LLZTO fillers, suggesting an improvement in thermal stability. Moreover, at temperatures as high as 550 °C, pure PVDF-HFP completely decomposes, while PH10L still retains undecomposed ceramic particles, demonstrating the beneficial effect of incorporating ceramic powders. TGA curves illustrated the initial thermal decomposition of PH0L at 428 °C, and both films exhibited commendable thermal stability, with no decomposition observed up to 400 °C (Figure S2) [27,35]. Simultaneously, the issue of thermal runaway in lithium batteries is a critically important safety concern. In this study, a combustion test was employed to investigate the flame retardancy of PH10L and a commercially available PE separator. As depicted in Figure 4a, the commercial PE separator was easily ignited, and by the third second, it was completely consumed in flames, with observable dripping of combustion by-products. In contrast, when a flame was introduced near PH10L, the shrinkage rate significantly decreased. If the flame source was removed, PH10L ceased combustion, as illustrated in Figure 4b. In addition, we have also provided video material of the combustion experiments. This suggests that the introduction of LLZTO resulted in a postponement of the separator’s combustion. Furthermore, the outstanding thermal stability of PH10L was substantiated by storing the separators at different ambient temperatures. As clearly observed in Figure 4c, a distinct trend emerged. With increasing temperature, the commercially available PE separator exhibited pronounced curling, whereas at 120 °C, PH10L maintained a high level of flatness. This visually demonstrates the thermal stability of PH10L, consistent with the earlier-discussed TG results and combustion tests.

### Electrochemical Performance

**Figure 5:**
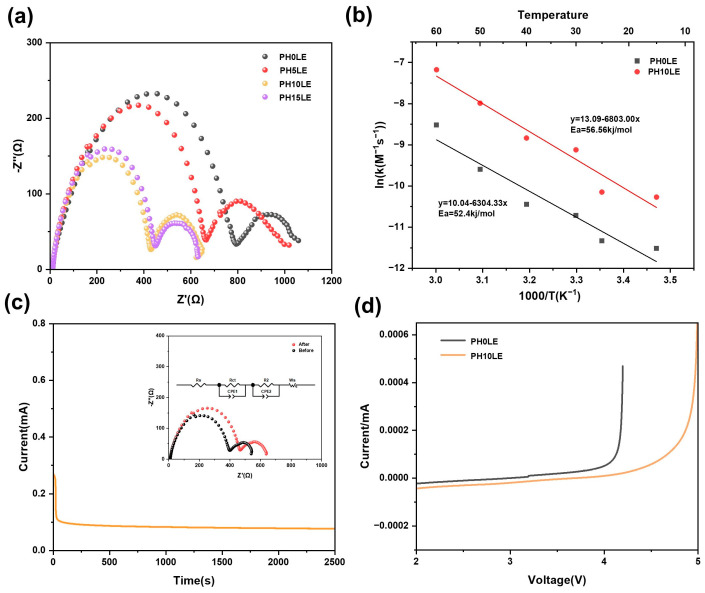
(**a**) EIS profiles of PH0LE to PH15LE, (**b**) The ionic conductivity of the PH10LE and PH0LE separator at different temperatures. (**c**) i-t curves of PH0LE and PH10LE at room temperature. (**d**) LSV of PH0LE and PH10LE at room temperature.

Systematic studies were conducted on the ionic conductivity of solid electrolytes using SS blocking electrodes. Figure 5a displays the EIS impedance spectra of PH*x*L CPE at room temperature. All curve shapes are consistent with a high-frequency region showing a downward-bent semicircular arc and a low-frequency region corresponding to a sloping straight line. The AC impedance spectra were fitted using an equivalent circuit through least squares fitting, yielding the bulk resistivity of the electrolyte. With the continuous increase in LLZTO content, the lithium-ion conductivity of the flexible electrolyte also increases. The ion conductivity of PH0LE is only 4.0 × 10^−5^ S cm^−1^. However, when the LLZTO content reaches 10%, the ion conductivity of PH10LE is maximized, reaching up to 1.21 × 10^−4^ S cm^−1^, which is much higher than that of PH0LE. This may be attributed to the addition of ceramic powders affecting the crystallinity of the polymer, resulting in an overall enhancement of the ionic conductivity [50]. Furthermore, the ionic conductivity of PH10LE and PH0LE was further tested at different temperatures, as depicted in the Arrhenius plot in Figure 5b. The temperature range for the FCPE electrolyte was set between 15 °C and 60 °C. From this, it can be observed that with an increase in temperature, the ionic conductivity of FCPE gradually increases. The ion conductivity of composite solid-state electrolytes in all-solid-state lithium-ion batteries without the addition of liquid electrolyte, as presented in Table S1 from recent reports, clearly indicates that the final results obtained in this study are outstanding [45,50]. Combining the analysis with Figure 5c, the increase in the amorphous region is also beneficial for the rapid migration of lithium ions. It elucidates the temporal evolution of current throughout the polarization process. The accompanying diagrams delineate the simulated equivalent circuit and impedance spectra, wherein R_s_, R_ct_, and R_2_ signify the resistances arising from the electrolyte bulk, grain boundaries, and both electrode–electrolyte interfaces, respectively. CPE_1_ and CPE_2_ are associated with constant phase elements at grain boundaries, LLZTO, and polymer interfaces. W_s_ characterizes the impedance encountered by reactants diffusing from the electrolyte bulk to the electrode reaction interface. The impedance response was meticulously computed using ZView software [37]. The 10 wt% LLZTO ensures that the amorphous region of the organic material is maximized without particle agglomeration, and the network structure itself is interconnected. At this concentration, 10 wt% LLZTO is sufficient to form a three-dimensional interconnected Li^+^ channel. Excessive LLZTO, on the other hand, can hinder ion transport, thus reducing the conductivity of Li^+^ [48]. Within this porous structure saturated with VEC precursors, the polymerized PVEC-based electrolyte serves as an ion conductor, intricately interacting with the 3D porous skeleton. This interaction results in the creation of a unique channel, facilitating the rapid transport of lithium ions. Lithium ions can not only be transported within the 3D net-like structure but also within the PVEC-based electrolyte, further enhancing lithium ion conductivity [51]. As shown in Figure 5d, the LSV measurements were measured with a voltage range of 0 to 4.8 V at room temperature. Compared to the PH0LE, the potential of PH10LE begins to decompose because of oxidation at as high as 4.7 V relative to Li^+^/Li which indicates that the PH10LE is very stable at high voltage.

**Figure 6:**
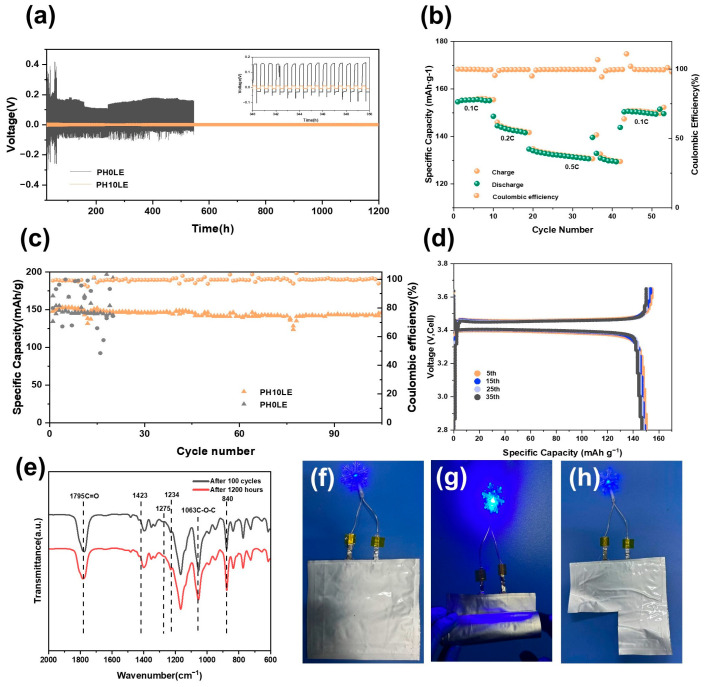
(**a**) Voltage profiles of Li|PH0LE|Li and Li|PH10LE|Li symmetrical cells at room temperature, and the inset in (**a**) shows magnified voltage profiles in an hour range of 340 h to 350 h. (**b**) Rate performance of Li/PH*x*LE/LFP (*x* = 0 or 10) coin cells at room temperature. (**c**) Cycling performance of Li/PH*x*LE/LFP (*x* = 0 or 10) coin cells at 0.1 C. (**d**) Charge–discharge curves of Li|PH10LE|LFP cell at different cycle numbers. FT-IR spectrum of (**e**) FCPE after 100 cycles of Li/FCPE/LFP and FCPE after 1200 h of cycling in Li/FCPE/Li. (**f**–**h**) Digital photograph of a blue LED light illuminated by an Li/FCPE/LFP pouch cell.

Lithium plating/stripping experiments were conducted in a symmetrical cell to demonstrate the interface stability of FCPE on the lithium metal. As shown in Figure 6a, the voltage-time curve indicates that the battery with PH10LE CPE can maintain stability at a current density of 0.1 mA cm^−2^, remaining stable even after 1200 h of cycling. In contrast, the battery using PH0LE CPE experiences a short circuit within 600 h (Figure 6a). Furthermore, compared to other lithium batteries, the polarization voltage of the Li|PH10LE|Li lithium battery is only 10 mV. These results suggest that this mesh-like FCPE is more effective in suppressing lithium dendrite growth and relatively regulating lithium deposition.

To further illustrate the advantages of the 3D net-like flexible composite electrolyte, a solid-state lithium metal battery was constructed with LFP as the cathode and its electrochemical performance was tested. In Figure 6b, it depicts the reversible charge–discharge capacities of the lithium metal battery at different rates (0.1 C–0.5 C). The stable capacities of the Li|PH10LE|LFP battery at 0.1 C, 0.2 C, and 0.5 C are 155.8, 146.3, and 136.7 mA h g^−1^, respectively. Upon switching back to 0.1 C, the capacity can recover to 152.1 mA h g^−1^, demonstrating outstanding rate capability. Figure 6c compares the long-term cycling performance and corresponding coulombic efficiency of the two batteries at 0.1 C. All-solid-state batteries assembled with PH0LE are shorted directly after the 20th cycle. In contrast, the Li|PH10LE|LFP battery achieves over 100 reversible electrochemical cycles and maintains a high specific capacity of 148.5 mA h g^−1^. Figure 6d shows the charge–discharge curves of the Li|PH10LE|LFP battery at different cycles at 0.1 C. From the 5th to the 35th cycle, the curves remain smooth, and the capacity stays relatively stable, indicating the absence of side reactions within the battery.

To validate the practical performance of this approach, a Li/FCPE/LFP pouch cell was assembled. After charging the pouch cell at a rate of 0.1 C to full capacity, no swelling phenomenon occurred. As shown in Figure 6e–h, at room temperature, the pouch cell was able to power a blue LED light normally. Furthermore, even when the pouch cell underwent arbitrary bending and shearing, it continued to supply power to the blue LED light without affecting its normal operation. This substantiates and underscores the extensive potential application of FCPE as elucidated in the study, indicating its promising utilization in flexible wearable electronic devices. In order to elucidate that no side reactions occur in the formation of the SEI (solid electrolyte interface) film during the battery cycling process in any component of the solid-state electrolyte, infrared tests were conducted on the FCPE of Li/FCPE/LFP after 100 cycles and Li/FCPE/Li after 1200 h of cycling (Figure 6e). The test outcomes reveal negligible deviation in characteristic peaks, signifying the ongoing stability of the solid electrolyte diaphragm structure throughout the cycling process. This further substantiates the cyclic stability of the solid electrolyte. The pouch cell assembled using PH10LE also exhibits excellent performance, as shown in Figure S3, achieving a capacity retention rate of 153.51 mA h g^−1^ at 0.1 C. This promising result suggests potential applications in future wearable electronic devices.

Based on the findings presented in the research, the augmentation mechanisms attributed to FCPE in enhancing lithium battery performance can be distilled into several key facets. First, ceramic particles enveloped within PVDF-HFP, culminating in the establishment of a robust PVDF-HFP/LLZTO interface and provide sufficient mechanical strength. The resulting three-dimensional network structure serves to intricately interconnect the lithium-ion transport interface. Second, within the 3D net-like structure, the ion conductor PVEC-based electrolyte permeates, adeptly gathering lithium ions along the pore walls. This not only amplifies ion conductivity but also benefits from the advantageous properties of VEC monomers, thereby fortifying Li^+^ ion conductivity and antioxidation capabilities. Complemented by a passive layer founded on LiF, the entire system exhibits commendable stability, both in the context of Li-metal interactions and overall operational endurance [49]. 

## 4. Conclusions

In summary, a universal and straightforward method has been developed for preparing a netlike flexible composite electrolyte. The results indicate that the 3D active nano-fillers in the PVDF-HFP-based CPE exhibit high thermal stability and outstanding Li^+^ conductivity. After filling the network with a PVEC-based electrolyte, the ion conductivity further increases to 1.21 × 10^−4^ S cm^−1^ at 25 °C. Moreover, the flexible composite electrolyte membrane creates a 3D net-like structure with multiple rapid pathways for Li^+^ that effectively control Li^+^ flux, leading to uniform lithium deposition. Consequently, the symmetrical lithium cells exhibited remarkable stability when using the FCPE, while the assembled Li/FCPE/LFP battery showcased exceptional cycling performance. FT-IR spectroscopy analysis of the solid electrolyte membrane post-cycling reveals that its structure remains stable, further confirming the structural stability of FCPE. This work introduces a novel strategy for advancing the enhancement of flexible all-solid-state batteries.

## Data Availability

Data are contained within the article or Supplementary Materials.

## Supplementary Materials

The following supporting information can be downloaded at: https://www.mdpi.com/article/10.3390/polym16050565/s1, Figure S1: The FT-IR spectrum of PVEC-based electrolyte, Figure S2: The TGA curve of PH0Land PH10L, Figure S3: Charge–discharge curves of Li|PH10LE|LFP pouch cell at 0.1 C, Table S1: Ionic conductivity in all-solid-state lithium-ion batteries have been reported.
