# Supplementary material for: Flexible Composite Electrolyte Membranes with Fast Ion Transport Channels for Solid-State Lithium Batteries
Source: Polymers (Basel). 2024 Feb 20;16(5):565. doi: 10.3390/polym16050565 (PMC10934709; doi:10.3390/polym16050565)
Supplement: Supplementary file 1 [file polymers-16-00565-s001.zip › polymers-2838703-supplementary.pdf]

# Supporting Information

# Flexible Composite Electrolyte membranes with Fast Ion Transport Channels for Solid-State Lithium Batteries

Xiaojun Ma[1], Dongxu Mao[1], Wenkai Xin[1], Shangyun Yang[1], Hao Zhang[1], Yanzhu Zhang[1], Xundao Liu[1], Dehua Dong[2], Zhengmao Ye [1,*], Jiajie Li [1,*]

1. School of Materials Science and Engineering, University of Jinan, Jinan, 250022, P. R. China

2. Department of Chemical and Biological Engineering, Monash University, Clayton, VIC 3800, Australia

* To whom correspondence should be addressed. E-mail: mse_lijj@ujn.edu.cn; mse_yezm@ujn.edu.cn; Tel: (+86) 531-89736011. Fax: (+86) 531-89736011.

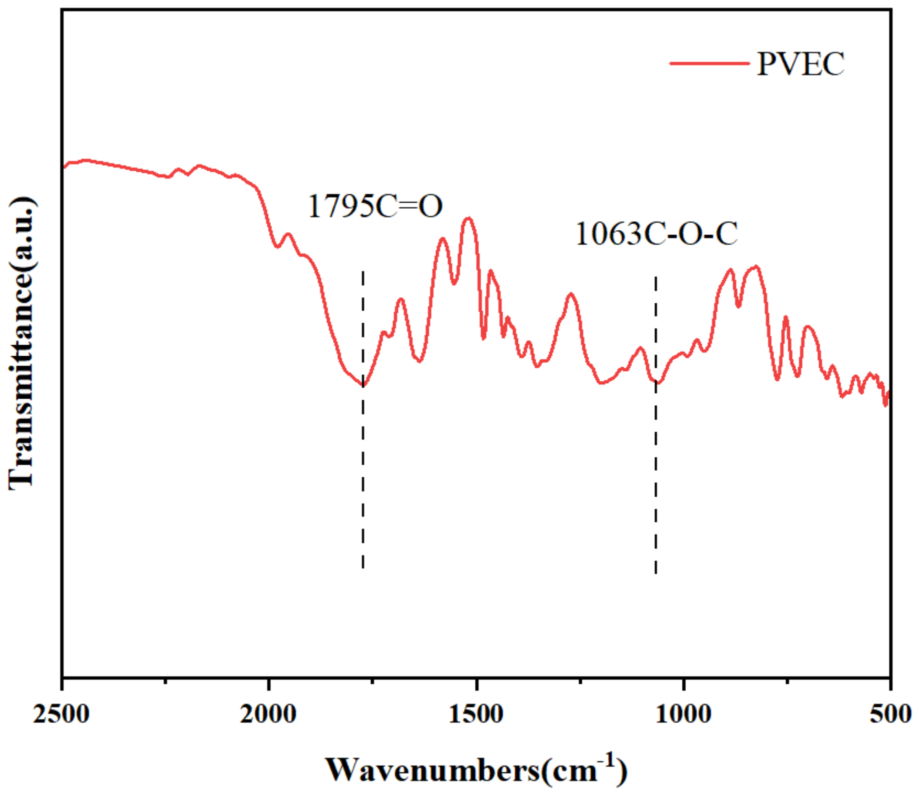


Figure S1: The FT-IR spectrum of PVEC-based electrolyte

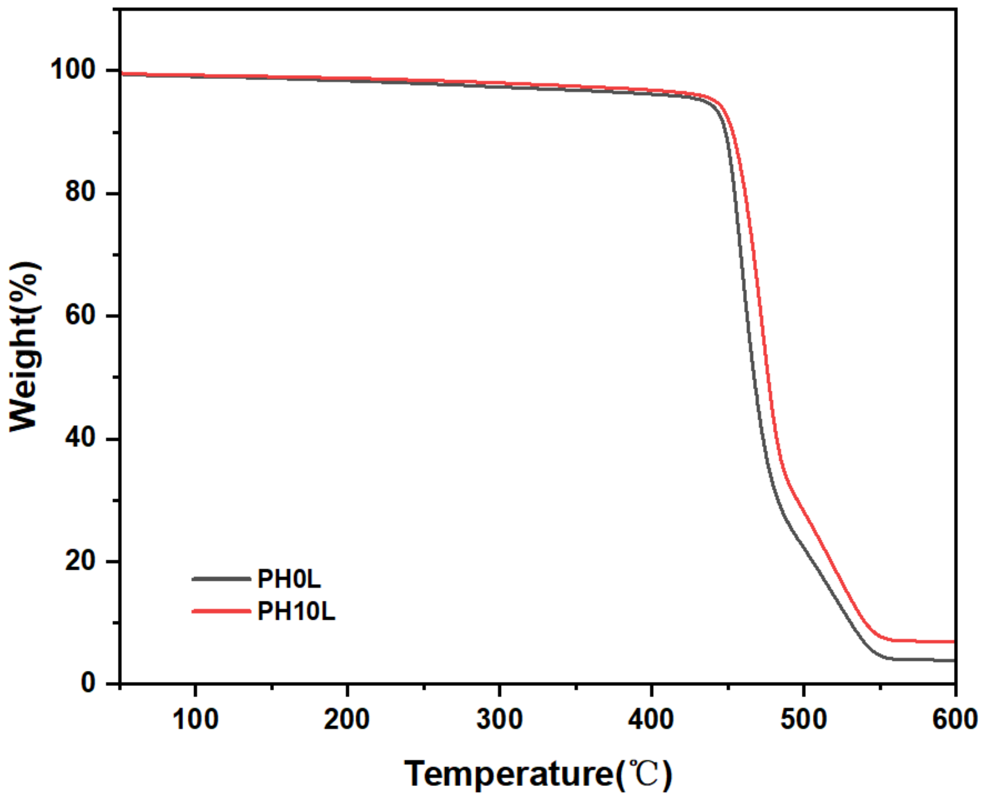


Figure S2: The TGA curve of PH0Land PH10L

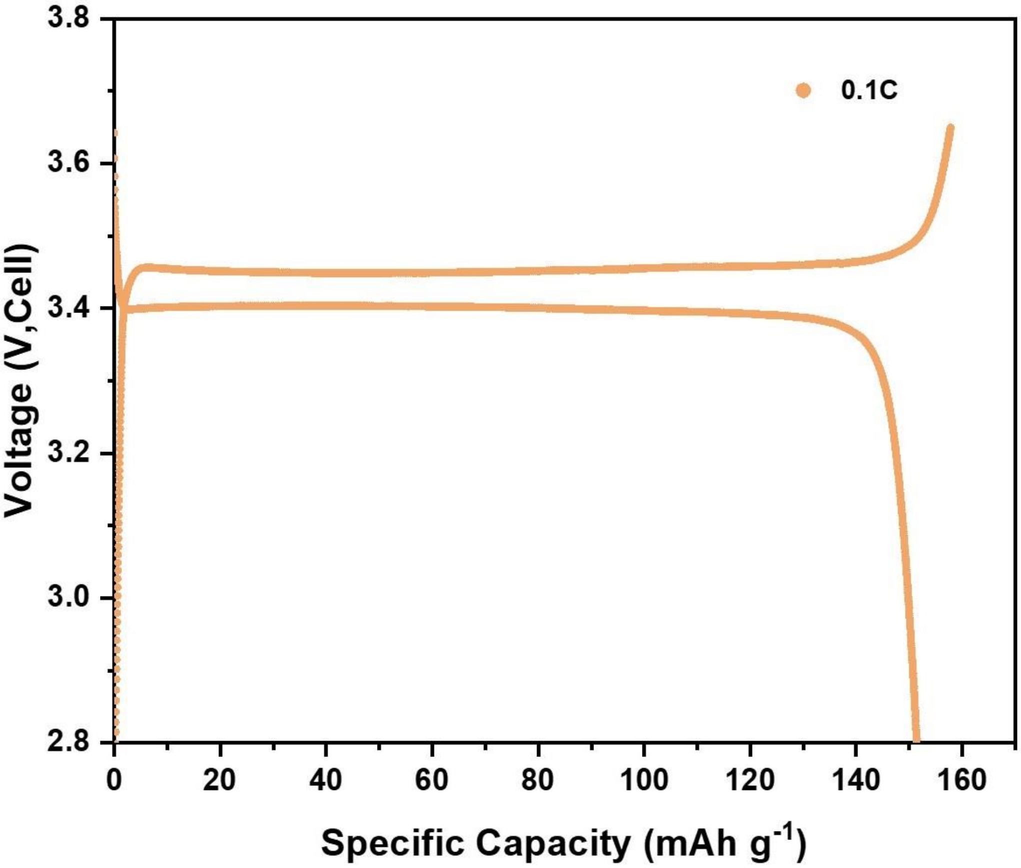


Figure S3: Charge-discharge curves of Li|PH10LE|LFP pouch cell at 0.1C

Table S1 Ionic conductivity in all-solid-state lithium-ion batteries has been reported

| CPEs | Ionic conductivity (free liquid electrolyte) | Reference |
|---|---|---|
| PVDF-HFP\|LLZO | 1.23 × 10−6 S cm−1 | Nano Energy 45 (2018) 413–419[1] |
| PVDF\|LLZTO@PDA | 1.52 × 10−4 S cm−1 | Adv. Energy Mater. 2023, 2204377[2] |
| PVDF\|LLZTNO | 1.05 × 10−4 S cm−1 | Journal of Power Sources 564 (2023) 232849[3] |
| PVDF-80wt%LLZTO-CSE | 1.06 × 10−5 S cm−1 | Ionics (Kiel) 2021, 27, 3339-3346.[4] |
| F-CSE | 1.01 × 10−4 S cm−1 | Adv. Funct. Mater. 2021, 2010611[5] |
| PH10LE | 1.21× 10−4 S cm−1 | this work |

1. Zhang, W.; Nie, J.; Li, F.; Wang, Z. L.; Sun, C. A durable and safe solid-state lithium battery with a hybrid electrolyte membrane. *Nano Energy* **2018,** *45*, 413-419.
2. Xu, Y.; Wang, K.; Zhang, X.; Ma, Y.; Peng, Q.; Gong, Y.; Yi, S.; Guo, H.; Zhang, X.; Sun, X.; Gao, H.; Xin, S.; Guo, Y. G.; Ma, Y. Improved Li-Ion Conduction and (Electro)Chemical Stability at Garnet-Polymer Interface through Metal-Nitrogen Bonding. *Adv Energy Mater* **2023,** *13*, 2204377.
3. Song, X.; Zhang, T.; Huang, S.; Mi, J.; Zhang, Y.; Travas-Sejdic, J.; Turner, A. P.; Gao, W.; Cao, P. Constructing a PVDF-based composite solid-state electrolyte with high ionic conductivity $Li_{6.5}La_3Zr_{1.5}Ta_{0.1}Nb_{0.4}O_{12}$ for lithium metal battery. *J Power Sources* **2023,** *564*, 232849.
4. Liu, H.; Li, J.; Feng, W.; Han, G. Strippable and flexible solid electrolyte membrane by coupling $Li_{6.4}La_3Zr_{1.4}Ta_{0.6}O_{12}$ and insulating polyvinylidene fluoride for solid state lithium ion battery. *Ionics (Kiel)* **2021,** *27*, 3339-3346.
5. Li, X.; Cong, L.; Ma, S.; Shi, S.; Li, Y.; Li, S.; Chen, S.; Zheng, C.; Sun, L.; Liu, Y.; Xie, H. Low Resistance and High Stable Solid–Liquid Electrolyte Interphases Enable High-Voltage Solid-State Lithium Metal Batteries. *Adv Funct. Mater.* **2021,** *31*, 2010611.
